# Association of ambient temperature and diurnal temperature range exposure during adolescence with adult IIEF-5 and PEDT scores: a cross-sectional study in China

**DOI:** 10.3389/frph.2026.1955542

**Published:** 2026-10-16

**Authors:** Weiqian Zhang, Rui Qu, Jiayu Wang, Jingxuan Wang, Feng Zhang, Yiqiu Wei, Zihan Guo, Yan Zhang, Jue Liu, Tailang Yin, Dongdong Tang

**Affiliations:** 1 Reproductive Medicine Center, Renmin Hospital of Wuhan University, Wuhan, Hubei, China; 2 Department of Clinical Laboratory, Renmin Hospital of Wuhan University, Wuhan, Hubei, China; 3 Department of Epidemiology and Biostatistics, School of Public Health, Peking University, Beijing, China; 4 Institute for Global Health and Development, Peking University, Beijing, China; 5 Ministry of Education, Key Laboratory of Epidemiology of Major Diseases (Peking University), Beijing, China; 6 Department of Obstetrics and Gynecology, Reproductive Medicine Center, The First Affiliated Hospital of Anhui Medical University, Hefei, China; 7 NHC Key Laboratory of Study on Abnormal Gametes and Reproductive Tract (Anhui Medical University), Hefei, China; 8 Key Laboratory of Population Health Across Life Cycle (Anhui Medical University), Ministry of Education of the People’s Republic of China, Hefei, China

**Keywords:** adolescence, diurnal temperature range, erectile dysfunction, extreme temperature, male sexual function, premature ejaculation

## Abstract

**Background:**

Exposure to extreme temperatures and diurnal temperature range (DTR) has been linked to impaired male reproductive health, yet studies investigating the associations between such exposures during adolescence and adult male sexual function (MSF) remain limited.

**Methods:**

We analyzed data from 5,039 sexually active males across 31 provinces in China, drawn from a two-stage quota-sampled cross-sectional online survey conducted in 2023. Exposure periods included all adolescence (10–19 years), early adolescence (10–14 years), and late adolescence (15–19 years). Adolescent temperature and DTR exposures were retrospectively estimated using participants' current adult residential addresses. Generalized additive models and hierarchical linear models were employed to assess the associations between temperature and DTR exposure and self-reported MSF, measured by the Premature Ejaculation Diagnostic Tool (PEDT) and the International Index of Erectile Function-5 (IIEF-5).

**Results:**

Generalized additive models suggested U-shaped and J-shaped trends for ambient temperature and DTR with PEDT scores, respectively, and formal tests confirmed significant departure from linearity. Exposure to extremely hot temperatures (> 90th percentile) during early adolescence was associated with higher PEDT scores (*β*: 0.67, 95% CI: 0.24–1.10) compared to moderate temperatures. Higher DTR exposure (>80th percentile) during all adolescence (*β*: 0.75, 95% CI: 0.29–1.22), early adolescence (*β*: 0.75, 95% CI: 0.28–1.21), and late adolescence (*β*: 0.48, 95% CI: 0.00–0.96) was also associated with higher PEDT scores. No significant associations were observed between temperature or DTR exposure and IIEF-5 scores.

**Conclusions:**

Our study provides preliminary epidemiological evidence that adolescent exposure to extremely hot temperatures and higher DTR is associated with higher PEDT scores in adulthood, with no association observed for IIEF-5 scores.

## 1 Introduction

Male sexual dysfunction (MSD) represents a critical public health issue that profoundly affects the quality of life of both patients and their partners, leading to detrimental consequences for male reproductive health and psychological well-being (1). Premature ejaculation (PE) and erectile dysfunction (ED) are the most prevalent manifestations of MSD (2). ED is a highly common condition, and approximately 30%–40% of adult men experience moderate to severe forms of the disorder (3, 4). The prevalence of PE is estimated to range from 20% to 30% across all age groups of men (5–7).

Male sexual development and maturation occur primarily during adolescence. According to the World Health Organization (WHO), adolescence spans from ages 10–19, representing the transition from childhood to adulthood (8). A hallmark of this developmental stage is the maturation of the gonads, alongside the rapid development of reproductive organs and secondary sexual characteristics. Simultaneously, significant changes occur in brain function, psychology, and behavior (9, 10). This phase is crucial for establishing long-term health and well-being. For instance, marijuana use during adolescence has been linked to an increased risk of memory deficits in adulthood (11), whereas adopting a healthy lifestyle during adolescence could prevent fatty liver (12). Environmental exposures during adolescence have been associated with altered reproductive development and with male reproductive health in adulthood. However, the association between environmental exposures during adolescence and adult male sexual function (MSF) has not been well examined.

The escalating trend of global warming is exacerbating the frequency and intensity of extreme weather events. China Climate Change Blue Book (2026) reports that from 1961 to 2025, China's annual average temperature increased by 0.31 °C per decade, exceeding the global average warming rate over the same period. This warming is particularly pronounced in rapidly urbanizing areas, where the urban heat island effect further amplifies the increase in surface air temperature (13). In terms of the male reproductive system, recent studies have reported associations between both extremely high and low ambient temperatures and sperm quality (14, 15). Additional research has also reported associations between temperature variability and both the quality and quantity of sperm (16, 17). Moreover, some studies have investigated seasonal fluctuation in male sexual dysfunction, with increased Google searches related to ED during winter months suggesting a potential seasonal variation in its prevalence (18, 19).

Adolescent development results from the intricate interplay of genetic predispositions and environmental factors. Due to their developmental physiology, adolescents may be more vulnerable to environmental hazards. Studies have reported that exposure to radiation or chemicals during adolescence is associated with altered testicular function in adulthood (20). Exposure to agents such as lead or endocrine disruptors during this developmental period has been associated with altered male hormone levels and reduced semen quality (21, 22). While the association between temperature exposure and physiological functions is well-documented, no studies have specifically investigated the association between extreme ambient temperatures or diurnal temperature fluctuations during adolescence and adult male sexual function. This gap in research is particularly relevant in the Chinese context, where rapid urbanization and high population density may amplify such environmental health concerns. This study aims to address this gap by examining the association between climate-related exposures and male sexual health across developmental stages.

Hence, we conducted a multi-province observational study to examine the association between exposure to extreme temperatures and temperature variability during adolescence and MSF in adulthood. Our findings may provide preliminary epidemiological evidence on this association and may help inform future research on adolescent thermal exposure and male sexual health.

## 2 Methods

### 2.1 Participant recruitment

Through deploying an anonymous online survey on “Survey Star” (Changsha Ran Xing Technology, Changsha, China, https://www.wjx.cn/), a platform engaging over 6.2 million individuals, we received a total of 12,157 valid responses spanning all 31 provinces throughout China from July to August in 2023. To ensure the sample accurately reflected the national demographics, a two-stage quota sampling strategy was employed. Stage 1 (Geographic stratification by region) was based on the population distribution reported in the China Health Statistics Yearbook (https://www.yearbookchina.com). We divided China into three major economic regions: Eastern (42.1% of national population), Central (29.7%), and Western (28.1%). Quotas were predetermined for each region proportional to their population share. In Stage 2 (Quota sampling by sex within each region), we further applied a quota based on the national sex ratio (male/female) as reported in the same yearbook within each geographic stratum. Invitations were distributed through the platform's participant pool across all 31 provinces, with a detailed invitation letter explaining the study's theme and objectives and providing a link to the online questionnaire. To ensure data accuracy, each account could only submit the questionnaire once after all questions had been completed, and responses were screened for logical inconsistencies (e.g., contradictory answers to related items) and implausibly short or long completion times. Responses failing these checks were excluded. The following were the inclusion criteria of this study: (1) Age range is 20–40. (2) No significant physical illness. (3) The individuals are married. After applying the inclusion and exclusion criteria, the final sample comprised 5,039 sexually active males aged 20–40 years from 31 provinces in China (Figure 1). Quotas by region and sex were applied during recruitment, but the sample should not be considered nationally representative.

**Figure 1:**
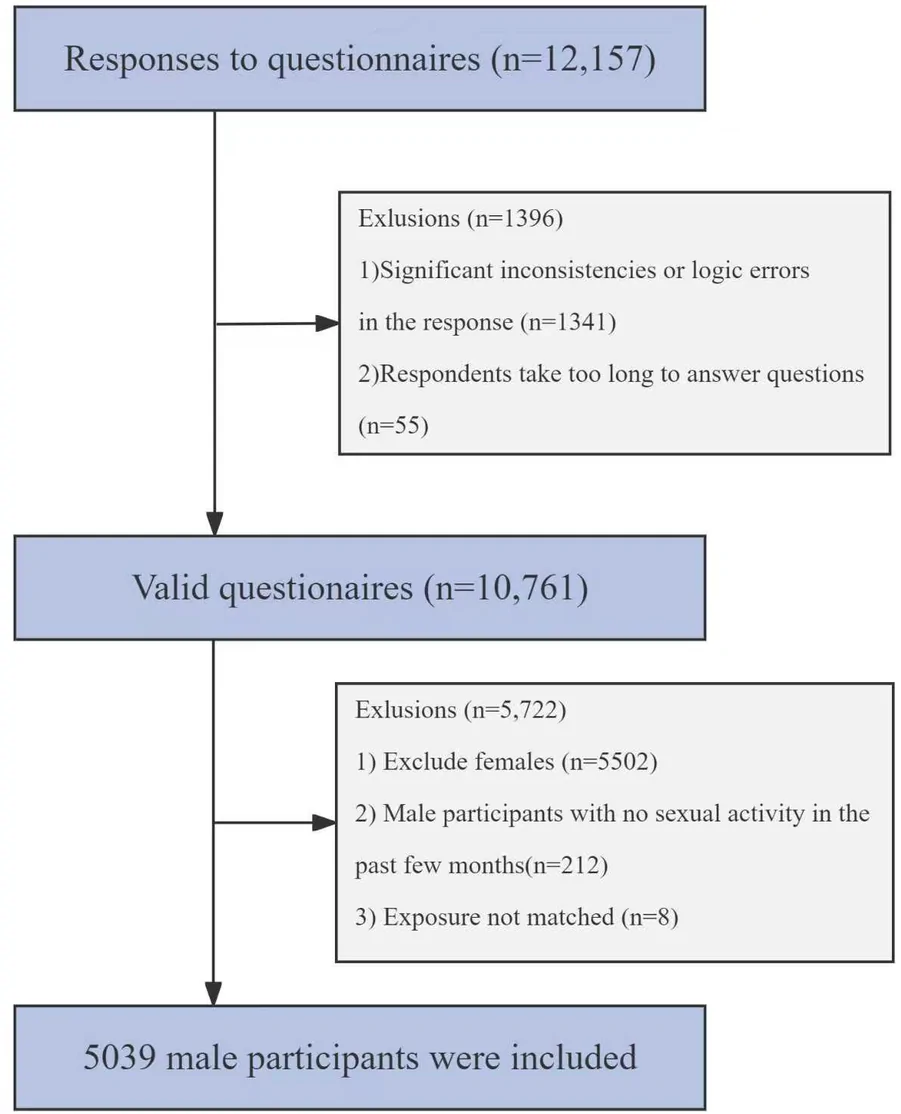
Diagram outlining the criteria for excluding participants in this study.

### 2.2 Evaluation of MSF

We employed the International Index of Erectile Function-5 (IIEF-5) and Premature Ejaculation Diagnostic Tool (PEDT) to assess MSF (23). The IIEF-5 questionnaire comprises four items from the erectile function subscale and one item from the sexual intercourse satisfaction subscale (24). Responses to each item are rated on a scale from 1 to 5, with a score of “0” representing no sexual stimulation or contact. The individual scores are summed to produce a total score, with higher scores reflecting better erectile function and greater satisfaction with sexual activity. Several previous studies have predominantly utilized the Chinese version of the IIEF-5 scale to assess erectile function (25, 26). The PEDT is a self-reporting instrument to assess premature ejaculation in men, encompassing five domains: control over ejaculation, frequency of early ejaculation, minimal sexual stimulation, emotional distress, and interpersonal issues. Each domain is rated on a scale from 0 to 4, with higher sum scores indicating a greater likelihood of PE (27). The Chinese version of the PEDT has undergone linguistic validation, confirming its validity as a trustworthy tool for impartially measuring PE (28). In this study, the IIEF-5 and PEDT were used as self-reported screening instruments for sexual function; they do not constitute a clinical diagnosis of erectile dysfunction or premature ejaculation.

### 2.3 Environmental exposure

The WHO defines adolescence as being between the ages of 10 and 19, while the Lancet Commission on Adolescent Health and Wellbeing further categorizes this age range into early adolescence (10–14 years) and late adolescence (15–19 years) (29). Therefore, we chose all adolescence, early adolescence, and late adolescence as exposure periods. Daily mean temperature, maximum temperature, and minimum temperature were obtained via the National Oceanic and Atmospheric Administration's global meteorological website (https://www.ncei.noaa.gov/data/global-summary-of-the-day/archive/). Particles less than 2.5 micrometers in diameter (PM_2.5_), particles less than 10 micrometers in diameter (PM_10_), sulfur dioxide (SO_2_), carbon monoxide (CO), nitrogen dioxide (NO_2_), and ozone (O_3_) were collected via the National Urban Air Quality Real-time Publishing Platform (https://air.cnemc.cn:18007/). Normalized difference vegetation index (NDVI), an assessment of green exposure, was derived from the Moderate Resolution Imaging Spectroradiometer (MODIS) on satellites launched by the National Aeronautics and Space Administration of the United States of America (https://modis.gsfc.nasa.gov/data/dataprod/mod13.php). MODIS provides spatially resolved NDVI once every month. The diurnal temperature range (DTR) was defined as the difference between the maximum and minimum temperatures within a single day and was used as an indicator of daily temperature fluctuation. Based on participants' current adult residential addresses and the locations of meteorological monitoring stations, we retrospectively estimated each subject's historical exposure to temperature and DTR during adolescence using the inverse distance weighting (IDW) interpolation approach. Subsequently, exposure levels within the designated exposure period were calculated as the average of the daily exposure levels over this timeframe.

To examine the association between extreme ambient temperatures and IIEF-5 and PEDT scores, we divided mean temperature into three groups: extremely low temperature (<10th percentile), moderate temperature (the 10th–90th percentile), and extremely high temperature (>90th percentile), with moderate temperature (the 10th–90th percentile) as the reference (30). In the main analysis, the 10th and 90th percentile thresholds were calculated across the entire national study population for each exposure period. For the north and south subgroup analyses, region-specific thresholds were calculated separately within northern (latitude ≥ 33°N) and southern (latitude <33 °N) China. The corresponding cut-off values are reported in Supplementary Table S1. Following previous studies, DTR was divided into quintiles (Q1–Q5), with the first quintile group (Q1: <20th percentile) serving as the reference group (16).

### 2.4 Covariates

Potential confounding variables were selected using a directed acyclic graph (Supplementary Figure S1) and were included as covariates to adjust for confounding in the subsequent analyses. Based on previous literature, the encompassed environmental and personal factors were as follows (31–37). (1) Demographics: age, population, residence, region, and income. (2) Lifestyle: body mass index (BMI), smoking, and alcohol consumption status. (3) Personal characteristics and psychological status: personality and depression score. (4) Health status: health constitution and underlying disease. Underlying diseases known to be associated with sexual dysfunction, such as cardiovascular disease, diabetes, and hypertension, were specifically considered. Temperature, NDVI, and air pollutants (PM_2.5_, PM_10_, CO, NO_2_, O_3_, and SO_2_) were averaged over the 1 month preceding the survey and included as covariates.

For personality assessment, we used a simple self-reported question in which participants were asked to categorize themselves as either “extroverted” or “introverted” based on their general social preferences and energy patterns in interpersonal relationships. This categorical approach was adopted for its practicality and efficiency in large-scale survey administration. In the assessment of depressive symptoms, we used the widely validated Patient Health Questionnaire-9 (PHQ-9). This scale consists of 9 items, and participants rated the frequency of each depressive symptom over the past two weeks on a 4-point scale (0 = “not at all” to 3 = “nearly every day”). The total score ranges from 0 to 27, with established cut-off scores indicating: none or minimal (0–4), mild to moderate (5–14), and moderate to severe (15–27) depressive symptoms.

### 2.5 Statistical analyses

Detailed demographic characteristics were presented as *n* (%), mean ± standard deviation, and median with quartiles. We used the Mann–Whitney test and the Kruskal–Wallis test to make group comparisons. Subsequently, after controlling for covariates, Generalized Additive Models (GAMs) were employed to accommodate potential non-linear relationships between mean temperature, DTR, and IIEF-5 and PEDT scores. The reference values used to fit the GAM curves were 16.03 °C, 16.16 °C, and 16.10 °C for early, late, and all adolescence temperature, respectively, and 9.52 °C, 9.65 °C, and 9.62 °C for early, late, and all adolescence DTR, respectively. Departure from linearity was tested by comparing a model with a linear term for the exposure to a model with a penalized smooth term [*s*(*x*, *k* = 3)] using an *F*-test [anova() function in the mgcv package]. For DTR models, temperature during the corresponding exposure period was included as a covariate. Hierarchical linear models were utilized to examine the association between extreme temperatures and DTR on IIEF-5 and PEDT scores. When investigating the association between DTR and IIEF-5 and PEDT scores, the temperature of that particular exposure period was included as a covariate in the model (38–41). *β* and 95% confidence interval (CI) were employed to quantify the magnitude of the association between temperature, DTR, and IIEF-5 and PEDT scores. The primary hypothesis was that adolescent exposure to extreme heat and higher DTR is associated with higher PEDT scores. Analyses involving IIEF-5 scores, the cold-exposure category, and subgroup comparisons by age and region were considered secondary and exploratory. No correction for multiple comparisons was applied to these exploratory analyses, and their results should therefore be interpreted with caution.

In addition, subgroup analyses were conducted to explore whether associations differed across age and geographic subgroups. Age subgroups were defined by individual characteristics (age ≤30 or >30). We utilized the Qinling-Huai River at approximately 33 °N latitude to demarcate southern and northern China (42). Sensitivity analyses were performed by (1) excluding adult post-exposure covariates (BMI, depression, underlying disease, smoking, and alcohol consumption status) and (2) adjusting for the mean air pollutants (PM_2.5_ and PM_10_) and NDVI during each exposure period during adolescence.

All analyses were performed using R 4.3.2. A *P*-value <0.05 was deemed statistically significant in the two-sided statistical tests.

## 3 Results

### 3.1 General characteristics of study participants

Our study ultimately enrolled 5,039 men from 31 provinces in China. The detailed demographic characteristics of the participants are presented in Table 1. The median (interquartile range, IQR) of the IIEF-5 scores and PEDT scores were 21.00 [18.00, 23.00] and 10.00 [8.00, 13.00], respectively. The age range of participants was 20–40 years, of whom 94.4% were Han Chinese and 92% lived in urban areas. In univariable comparisons (Table 1), lower BMI, presence of underlying disease, and moderate to severe depression were associated with lower IIEF-5 scores, whereas never smoking, never drinking alcohol, higher income, good constitution, and an extroverted personality were associated with higher IIEF-5 scores. Older age, presence of underlying disease, and moderate to severe depression were associated with higher PEDT scores, whereas never smoking, never drinking alcohol, good constitution, and an extroverted personality were associated with lower PEDT scores. These comparisons were univariable and should not be interpreted as evidence of independent associations. The mean temperatures for all, early, and late adolescence were 16.09 °C, 16.03 °C, and 16.16 °C, respectively, while the DTR during all, early, and late adolescence were 9.04 °C, 9.02 °C, and 9.06 °C (Supplementary Figure S2).

**Table 1:** Demographic characteristics and male sexual function scores of study subjects (*n* = 5,039).

Characters	*N* (%)	IIEF-5					PEDT				
		Mean ± SD	*P25*	*P50*	*P75*	*P* value	Mean ± SD	*P25*	*P50*	*P75*	*P* value
Age (Years)						<0.001*					<0.001*
20–24	1,069 (21.2)	19.55 ± 4.36	17	20	23	bc	10.54 ± 3.70	8	10	13	^c^ ^,^ ^d^
25–29	1,582 (31.4)	20.18 ± 3.71	18	21	23	^a^	10.68 ± 3.73	8	10	13	^d^
30–34	1,552 (30.8)	20.33 ± 3.52	19	21	23	^a^	10.95 ± 3.74	8	11	13	^a^
35–40	836 (16.6)	20.10 ± 3.49	18	21	23		11.37 ± 3.92	9	11	14	^a^ ^,^ ^b^
Population						0.04					0.59
H^a^n Chinese	4,758 (94.4)	20.11 ± 3.75	18	21	23		10.84 ± 3.77	8	10	13	
Non-Han Chinese	281 (5.6)	19.56 ± 4.11	17	20	23		10.95 ± 3.84	8	11	13	
BMI						<0.001*					0.638
<18.5	390 (7.7)	19.20 ± 4.26	17	20	22	^b^ ^,^ ^c^	10.81 ± 3.70	8	10	13	
18.5–24.49	3,399 (67.5)	20.24 ± 3.63	18	21	23	^a^ ^,^ ^d^	10.79 ± 3.74	8	10	13	
24.5–29.99	1,184 (23.5)	19.99 ± 3.91	18	21	23	^a^	11.01 ± 3.88	8	10	13	
≥30	66 (1.3)	18.68 ± 4.49	16	19	23		10.98 ± 4.14	8	10	13	
Smoking status						<00.001*					<0.001*
Never	2,326 (46.2)	20.26 ± 3.90	19	21	23	^b^ ^,^ ^c^	10.46 ± 3.76	8	10	13	^b^ ^,^ ^c^
Former	724 (14.4)	20.07 ± 3.53	18	21	23	^a^	11.02 ± 3.47	9	11	13	^a^
Current	1,989 (39.5)	19.87 ± 3.70	18	21	23	^a^	11.24 ± 3.85	8	11	14	^a^
Alcohol consumption status						<0.001*					<0.001*
Never	1,568 (31.1)	20.24 ± 3.99	18	21	23	^b^ ^,^ ^c^	10.26 ± 3.82	7	10	12	^b^ ^,^ ^c^
Former	523 (10.4)	19.84 ± 3.86	18	20	23	^a^	10.73 ± 3.66	8	11	13	^a^
Current	2,948 (58.5)	20.03 ± 3.64	18	21	23	^a^	11.18 ± 3.73	9	11	13	^a^
Education level						<0.001*					0.01
Secondary education or lower	447 (8.9)	19.15 ± 4.31	17	20	22	^b^ ^,^ ^c^	11.02 ± 3.89	8		13	^c^
Undergraduate level	4,025 (79.9)	20.12 ± 3.70	18	21	23	^a^ ^,^ ^c^	10.89 ± 3.77	8	10	13	^c^
Postgraduate level or higher	567 (11.3)	20.51 ± 3.74	19	21	23	^a^ ^,^ ^b^	10.39 ± 3.67	8	10	13	^a^ ^,^ ^b^
Income (Yuan/month)						<0.001*					0.02
<5,000	1,012 (20.1)	19.07 ± 4.52	17	20	22	^b^ ^,^ ^c^	10.82 ± 3.79	8	10	13	
5,000–10,000	2,751 (54.6)	20.15 ± 3.56	18	21	23	^a^ ^,^ ^c^	10.94 ± 3.73	8	11	13	^c^
>10,000	1,276 (25.3)	20.72 ± 3.40	19	21	23	^a^ ^,^ ^b^	10.66 ± 3.86	8	10	13	^b^
Residence						<0.001*					0.09
Urban areas	4,635 (92.0)	20.15 ± 3.70	18	21	23		10.82 ± 3.77	8	10	13	
Rural areas	404 (8.0)	19.27 ± 4.45	17	20	22		11.12 ± 3.82	8	11	13	
Region						<0.001*					0.49
Eastern Region	2,123 (42.1)	20.39 ± 3.62	19	21	23	^b^ ^,^ ^c^	10.82 ± 3.83	8	10	13	
Central Region	1,499 (29.7)	19.86 ± 3.87	18	21	23	^a^	10.93 ± 3.69	8	10	13	
Western Region	1,417 (28.1)	19.84 ± 3.87	18	21	23	^a^	10.81 ± 3.77	8	10	13	
Personality						<0.001*					<0.001*
Introverted	2,359 (46.8)	19.56 ± 3.94	18	20	23		11.39 ± 3.90	9	11	14	
Extroverted	2,680 (53.2)	20.53 ± 3.56	19	21	23		10.38 ± 3.59	8	10	12	
Constitution						<0.001*					<0.001*
Poor	250 (5.0)	16.92 ± 5.01	13	18	21	^b^ ^,^ ^c^	13.06 ± 4.26	10	13	16	^b^ ^,^ ^c^
Moderate	2,984 (59.2)	19.58 ± 3.67	18	20	22	^a^	11.38 ± 3.74	9	11	14	^a^ ^,^ ^c^
Good	1,805 (35.8)	21.33 ± 3.28	20	22	24	^a^	9.66 ± 3.40	7	9	12	^a^ ^,^ ^b^
Underlying disease condition						<0.001*					<0.001*
Absence	3,919 (77.8)	20.52 ± 3.58	19	21	23		10.40 ± 3.60	8	10	12	
Presence	1,120 (22.2)	18.54 ± 4.05	16	19	22		12.43 ± 3.93	10	12	15	
Depression_grade						<0.001*					<0.001*
None/Minimal	1,855 (36.8)	21.36 ± 3.19	20	22	24	^b^ ^,^ ^c^	9.41 ± 3.15	7	9	11	^b^ ^,^ ^c^
Mild to Moderate	2,814 (55.8)	19.59 ± 3.70	18	20	22	^a^	11.39 ± 3.69	9	11	14	^a^ ^,^ ^c^
Moderate to Severe	370 (7.3)	17.36 ± 4.61	14	18	21	^a^	13.94 ± 4.32	11	14	17	^a^ ^,^ ^b^
IIEF-5_score [median (IQR)]	21.00 [18.00, 23.00]										
PEDT_score [median (IQR)]	10.00 [8.00, 13.00]										

SD, standard deviation; BMI, body mass index; IQR, interquartile.

Data was presented as mean ± standard deviation, median, interquartile, and number (%).

* Between-group differences were examined through the Mann–Whitney test and Kruskal–Wallis test according to the data distribution.

a,b,c,d Indicates differences from groups 1, 2, 3, 4.

### 3.2 Associations of mean temperature and DTR with IIEF-5 and PEDT scores

First, we applied the GAM model to examine potential non-linear associations between mean temperature and DTR exposure and IIEF-5 scores and PEDT scores across each exposure period. As depicted in Figure 2, the fitted curves suggested a U-shaped trend between mean temperature and PEDT scores in every adolescent exposure period (Figures 2A–C), and a J-shaped trend between DTR and PEDT scores (Figures 2D–F). IIEF-5 scores showed a linear relationship with mean temperature across all exposure periods, whereas DTR showed a reverse J-shaped trend with IIEF-5 scores (Supplementary Figure S3).

**Figure 2:**
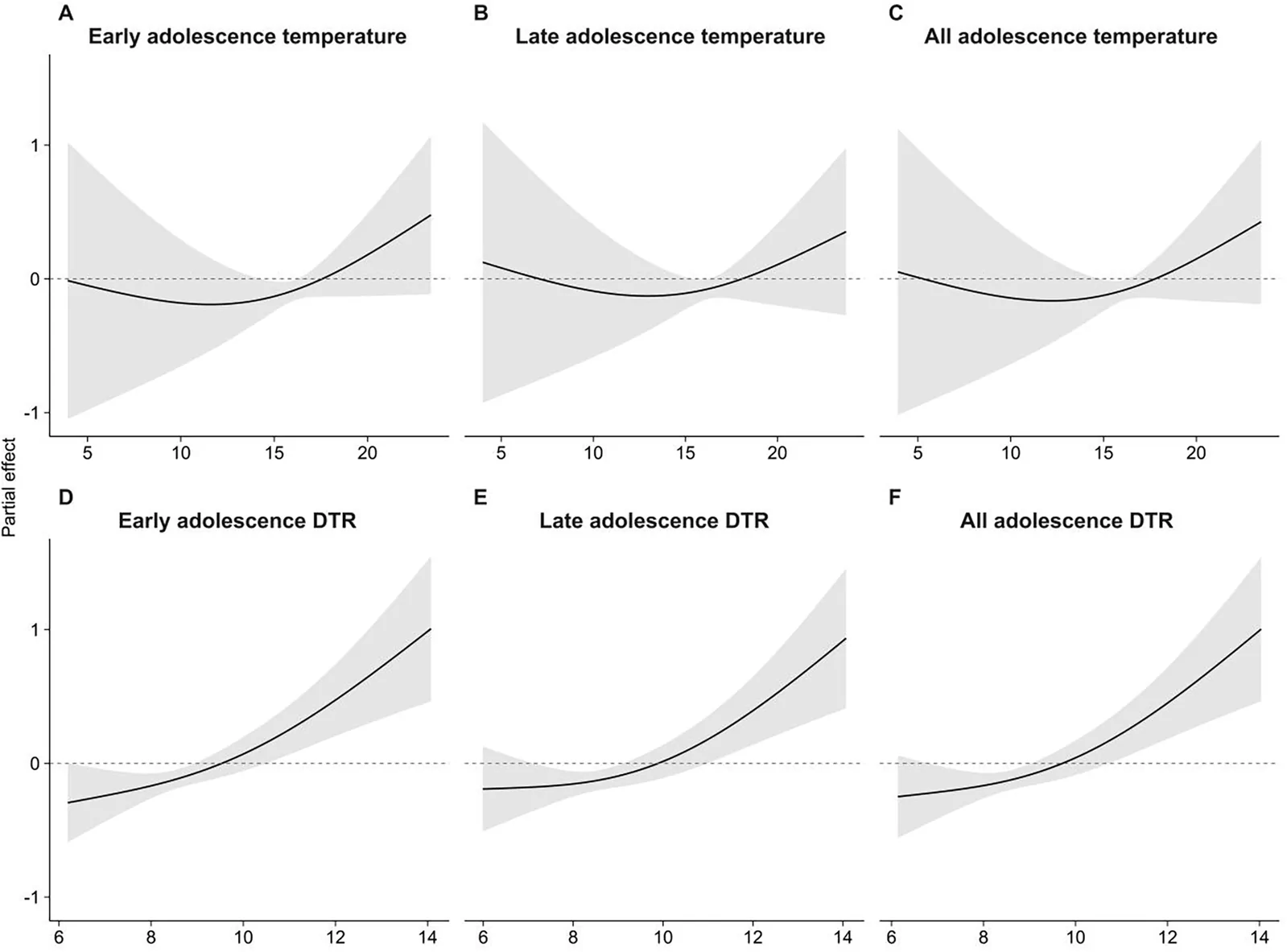
Curves of the associations between PEDT scores and average temperature and DTR exposure. The potential non-linear associations of temperature **(A–C)** and DTR **(D–F)** during all, early, and late adolescence with PEDT scores were quantified using multivariable GAM. The models were adjusted for age, population, residence, region, income, BMI, smoking status, alcohol consumption status, personality, constitution, underlying disease, depression score, temperature, NDVI and air pollutants (PM_2.5_, PM_10_, SO_2_, NO_2_, O_3_, and CO) during the 1 month before the survey, as well as temperature during the corresponding adolescence exposure period (only in DTR models). The reference exposure values were 16.03 °C, 16.16 °C, and 16.10 °C for early, late, and all adolescence temperature, respectively, and 9.52 °C, 9.65 °C, and 9.62 °C for early, late, and all adolescence DTR, respectively. The vertical axis indicates a partial effect on PEDT scores. Solid black lines correspond to points estimate of β of PEDT; Shaded areas correspond to 95% confidence intervals.

Formal nonlinearity tests confirmed significant departure from linearity for temperature exposure across all three periods (*P* = 0.02, 0.03, and 0.02 for early, late, and all adolescence, respectively) and for DTR exposure during late (*P* = 0.02) and all adolescence (*P* = 0.04), with a borderline result during early adolescence (*P* = 0.06) (Supplementary Table S2). However, the 95% confidence intervals of the fitted curves largely crossed the null, and these curves should therefore be interpreted as visual non-linear trends rather than statistically supported associations at specific exposure levels.

To further examine the associations between extreme temperature and DTR and IIEF-5 and PEDT scores, categorical analyses were performed after additional adjustment for covariates (Supplementary Table S3). Exposure to extremely high temperature (>90th percentile) in early adolescence was associated with higher PEDT scores (*β*: 0.67, 95% CI: 0.24–1.10; Figure 3A). Higher DTR exposure (quintile Q5) during all (*β*: 0.75, 95% CI: 0.29–1.22) and early (*β*: 0.75, 95% CI: 0.28–1.21) adolescence was also associated with higher PEDT scores (Figure 3C). Higher DTR exposure during late adolescence showed a borderline association with higher PEDT scores (*β*: 0.48, 95% CI: 0.00–0.96), which should be interpreted with caution given the number of comparisons performed (Figure 3C). No significant associations were observed between IIEF-5 scores and extreme temperature or DTR exposures during adolescence (*P* > 0.05; Figures 3B–D).

**Figure 3:**
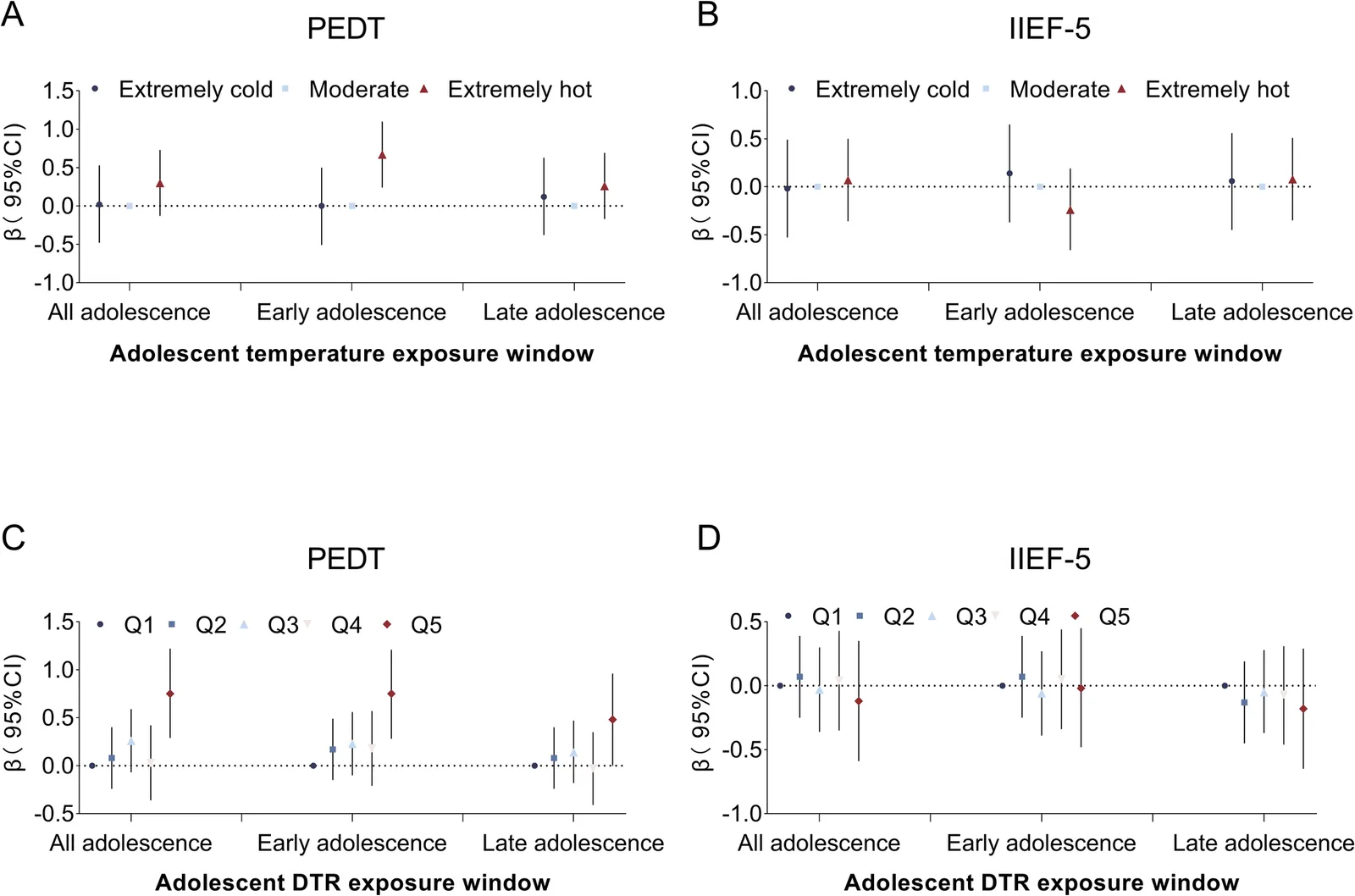
Mean difference (95% confidence interval) of male sexual function scores for extreme temperature and DTR exposure in all, early, and late adolescence. **(A)** 95% CI of PEDT scores for extreme temperature. **(B)** 95% CI of IIEF-5 scores for extreme temperature. **(C)** 95% CI of PEDT scores for DTR. **(D)** 95% CI of IIEF-5 scores for DTR. The models were adjusted for age, population, residence, region, income, BMI, smoking status, alcohol consumption status, personality, constitution, underlying disease, depression score, temperature, NDVI and air pollutants (PM_2.5_, PM_10_, SO_2_, NO_2_, O_3_, and CO) during the 1 month before the survey, as well as temperature during the corresponding adolescence exposure period (only in DTR models). The reference temperature was moderate (10th–90th percentile) and the reference DTR was Q1 (<20th percentile). The 10th and 90th percentile cut-offs were 10.78 °C and 22.61 °C for all adolescence, 10.71 and 22.54 °C for early adolescence, and 10.73 °C and 22.65 °C for late adolescence.

### 3.3 Subgroup and sensitivity analysis

Subgroup analyses were exploratory and not corrected for multiple comparisons. Table 2 presents the associations between mean temperature and DTR exposure and PEDT scores in different age subgroups during adolescence. Among participants aged 20–30 years, extremely high temperature exposure during early adolescence was associated with higher PEDT scores, whereas no statistically significant association was observed among those aged 31–40 years. Higher DTR exposure during early adolescence was associated with higher PEDT scores among males aged 20–30 years (*β*: 0.63, 95% CI: 0.03–1.23), and higher DTR exposure during early (*β*: 1.00, 95% CI: 0.25–1.76) and late (*β*: 0.82, 95% CI: 0.06–1.58) adolescence was associated with higher PEDT scores among males aged 31–40 years.

**Table 2:** Mean difference (95% confidence interval) of PEDT scores associated with extreme temperature and DTR exposure by age and area groups.

Subgroup	Adolescence periods	Temperature exposure					Quintile of DTR exposure		
		Extremely cold	Moderate	Extremely hot	Q1	Q2	Q3	Q4	Q5
Age, years									
20–30	Early adolescence	0.05 (−0.61,0.71)	0 (ref)	0.55 (0.01,1.10) *	0 (ref)	−0.11 (−0.52,0.30)	0.15 (−0.27,0.56)	0.24 (−0.25,0.72)	0.63 (0.03,1.23)*
	Late adolescence	0.08 (−0.57,0.73)	0 (ref)	0.41 (−0.14,0.97)	0 (ref)	−0.08 (−0.49,0.32)	0.12 (−0.29,0.53)	0.00 (−0.48,0.48)	0.51 (−0.08,1.11)
31–40	Early adolescence	−0.09 (−0.90,0.72)	0 (ref)	0.46 (−0.23,1.16)	0 (ref)	0.36 (−0.15,0.87)	0.33 (−0.20,0.86)	0.16 (−0.49,0.81)	1.00 (0.25,1.76)*
	Late adolescence	0.24 (−0.58,1.06)	0 (ref)	0.02 (−0.68,0.72)	0 (ref)	0.21 (−0.29,0.72)	0.21 (−0.31,0.74)	0.08 (−0.57,0.73)	0.82 (0.06,1.58)*
Area									
China North	Early adolescence	−0.81 (−1.65,0.03)	0 (ref)	0.27 (−0.37,0.91)	0 (ref)	−0.26 (−0.79,0.27)	−0.46 (−1.01,0.08)	0.23 (−0.40,0.85)	0.56 (−0.14,1.26)
	Late adolescence	−0.19 (−1.01,0.62)	0 (ref)	0.09 (−0.56,0.74)	0 (ref)	−0.04 (−0.56,0.48)	−0.30 (−0.84,0.25)	0.11 (−0.51,0.73)	0.58 (−0.11,1.26)
China South	Early adolescence	0.16 (−0.43,0.76)	0 (ref)	0.54 (0.04,1.04) *	0 (ref)	0.19 (−0.21,0.58)	0.21 (−0.20,0.62)	0.39 (−0.01,0.80)	0.50 (0.06,0.95)*
	Late adolescence	0.28 (−0.31,0.87)	0 (ref)	0.28 (−0.22,0.78)	0 (ref)	0.33 (−0.07,0.73)	0.08 (−0.33,0.50)	0.36 (−0.05,0.76)	0.52 (0.07,0.97)*

Model adjusted for age, population, residence, region, income, BMI, smoking status, alcohol consumption status, personality, constitution, underlying disease, depression score, temperature, NDVI and air pollutants (PM_2.5_, PM_10_, SO_2_, NO_2_, O_3_, and CO) during the 1 month before the survey, as well as temperature during the corresponding adolescence exposure period (only in DTR models). North and south subgroup analyses used region-specific percentile thresholds, calculated separately within each region. Subgroup analyses were stratified rather than formal interaction tests. The symbol “*” indicates statistical significance at *p* < 0.05. Q1–Q5: quintile.

When participants were categorized into north and south China, exposure to extremely high temperatures during early adolescence (*β*: 0.54, 95% CI: 0.04–1.04) and higher DTR exposure during early (*β*: 0.50, 95% CI: 0.06–0.95) and late (*β*: 0.52, 95% CI: 0.07–0.97) adolescence were associated with higher PEDT scores among males in south China. However, these subgroup analyses were stratified rather than formal interaction tests, and differences between subgroups should be interpreted with caution. Full results are presented in Table 2.

Our findings remained consistent in sensitivity analyses excluding adult post-exposure covariates (Supplementary Table S4) and in analyses additionally adjusting for air pollutants and NDVI during each adolescence exposure period (Supplementary Table S5).

## 4 Discussion

To the best of our knowledge, this study is one of the first large-scale investigations to examine the association between adolescent exposure to extreme temperatures and DTR and adult IIEF-5 and PEDT scores. Our findings suggest that exposure to extremely high temperatures and higher DTR during adolescence may be associated with higher PEDT scores. Given the retrospective cross-sectional design, causal relationships cannot be inferred. Nevertheless, the consistent patterns observed across multiple exposure periods and exploratory subgroup analyses suggest an association between adolescent thermal exposure and adult PEDT scores that warrants further investigation.

In the analysis of baseline clinical data, the median (interquartile range, IQR) of the IIEF-5 scores and PEDT scores are roughly in line with previous studies. A cross-sectional survey conducted across five regions of China, involving 1,239 men aged 18–60 years, demonstrated that the median scores for the IIEF-5 and PEDT were 20.00 (IQR: 13.00–22.00) and 9.00 (IQR: 5.50–13.00), respectively (43). From univariable comparisons (Table 1), moderate to severe depression, was associated with lower IIEF-5 scores and higher PEDT scores, consistent with previous research (44, 45); older age and underlying diseases were also associated with higher PEDT scores (46). Never smoking and never drinking alcohol were associated with higher IIEF-5 scores and lower PEDT scores, consistent with existing research (47, 48). However, these associations were univariable and should not be interpreted as independent protective effects. These factors may nonetheless be relevant to public health education as potential targets for prevention, although their independent contributions require confirmation in multivariable models.

Our findings are broadly consistent with previous studies on the male reproductive system. The association between adolescent mean temperature exposure and adult PEDT scores visually suggested a U-shaped pattern, and DTR visually suggested a J-shaped pattern. However, the 95% confidence intervals of the fitted GAM curves largely crossed the null across the exposure range, and the curves should therefore be interpreted as visual non-linear trends rather than statistically supported associations at specific exposure levels. In the categorical analyses, which grouped exposure ranges and had greater statistical power, extreme heat exposure was significantly associated with higher PEDT scores, whereas extreme cold exposure was not. Previous studies have reported associations between ambient temperature or temperature variability and sperm quality, with U-shaped or J-shaped exposure-response patterns (14, 17, 49–51). Experimental studies suggest that heat stress may be associated with sperm quality through oxidative stress, apoptosis, and DNA damage, whereas cold stress may act through apoptotic pathways (52–54). In addition, premature ejaculation has been linked to excessive sympathetic nervous system activity (55–57), and the maturation of reproductive behavior during adolescence involves brain remodeling and neural circuit activation (58, 59). Based on these findings, we speculate that exposure to extremely high temperatures and higher DTR during adolescence may act as chronic stressors on the developing sympathetic nervous system and sex-related neural circuits. However, our study did not measure hormones, pubertal development, autonomic function, semen quality, or neural outcomes, and the mechanisms proposed here therefore remain speculative. Future experimental and longitudinal studies are needed to test these hypotheses.

Intriguingly, previous studies have reported seasonal fluctuation in the onset of ED with the highest peak in winter (19). In contrast, our study found no association between IIEF-5 scores and exposure to extremely high temperatures and DTR during adolescence (Figure 3). The seasonal fluctuations in ED observed have been suggested to be partly related to diabetes, hypertension, and coronary vascular disease (CVD), and CVD events are known to peak during cold months. ED and CVD share a common pathophysiologic mechanism involving endothelial dysfunction and atherosclerosis, leading to decreased blood flow (18, 19). These seasonal patterns may therefore reflect concurrent adult exposures and underlying cardiovascular risk rather than adolescent temperature exposure. In our analysis, underlying diseases were included as covariates. However, no significant association between adolescent temperature or DTR exposure and IIEF-5 scores was observed in either the unadjusted or the adjusted models, suggesting that the null association for erectile function was not attributable to adjustment for underlying diseases.

In exploratory subgroup analyses, exposure to extremely high temperatures during early adolescence was associated with higher PEDT scores among males aged 20–30 years, whereas no statistically significant association was observed among those aged 31–40 years. This difference may partly reflect the imbalance in group sizes (61.1% vs. 38.9%), but no formal interaction test was performed, and this subgroup difference should therefore be interpreted with caution. The association between higher DTR exposure and PEDT scores was broadly similar in both age groups. In southern China, where extreme heat waves are more frequent, exposure to extremely high temperatures during early adolescence and higher DTR exposure during early and late adolescence were associated with higher PEDT scores. These subgroup findings were exploratory and require confirmation in independent studies.

Our findings suggest that extremely high temperatures and higher DTR during adolescence may be associated with adult PEDT scores. Unlike non-modifiable risk factors such as genetic predisposition (60, 61), climate-related exposures are potentially modifiable, which highlights the importance of further research in this area. In the context of global warming and rapid urbanization in China, the frequency and intensity of extreme heat events are increasing, which underscores the need for further research on their long-term health associations. However, given the observational design of our study and the retrospective reconstruction of adolescent exposures, our findings should be regarded as hypothesis-generating. They may provide a rationale for further investigation of adolescent thermal exposure and male sexual health, and prospective studies with longitudinal exposure assessment are needed before specific preventive recommendations can be made. If confirmed, such evidence could help inform future research on male reproductive health and the public health challenges posed by climate change.

Our study's strength emanates from the sizable cohort of sexually active Chinese men and the quantitative assessment of self-reported MSF through IIEF-5 and PEDT scores. Nevertheless, our research has several limitations.

First, a key limitation of this study is the absence of individual residential histories during adolescence. Adolescent temperature and DTR exposures were retrospectively estimated using participants' current adult residential addresses, which may introduce exposure misclassification. Because temperature and DTR can vary considerably within a province due to latitude, elevation, urbanization, topography, and local climate, and because we do not have information on participants' residential mobility between adolescence and adulthood, the direction and magnitude of this misclassification cannot be confidently determined. In addition, the retrospective nature of the exposure assessment precludes definitive causal inferences. Future prospective studies with longitudinal residential and exposure histories, tracking individuals from adolescence through adulthood, are needed to address this limitation and to confirm the causal relationship.

Second, several important confounding and exposure-measurement issues should be acknowledged. Several potentially important adolescent-life confounders were not available, including adolescent socioeconomic status, residence, family environment, puberty timing, childhood health, physical activity, and sleep. Moreover, some covariates were measured in adulthood and may represent post-exposure variables rather than adolescent confounders, which could lead to over-adjustment or collider bias; sensitivity analyses excluding these variables yielded consistent results (Supplementary Table S4). In addition, air conditioning, central heating, and clothing may make actual personal exposure inconsistent with ambient temperatures, and accurately determining personal exposure remains challenging. This may result in higher estimated exposure levels than actual levels. Residual confounding from unmeasured factors, such as exposure to hazardous pollutants, may also persist.

Third, environmental exposures were estimated using station-based IDW interpolation, which assumes that exposure varies primarily as a function of geographic distance. This approach may introduce spatial exposure error, particularly in regions with complex topography or sparse monitoring coverage, where elevation and other meteorological conditions can generate substantial spatial heterogeneity that is not captured by distance alone. Because the spatial error structure cannot be fully characterized, the direction and magnitude of this bias cannot be confidently determined.

Fourth, personality was assessed using a single self-reported binary item (extroverted vs. introverted) rather than a validated multi-dimensional tool. While this simplified approach was necessary for the large-scale survey setting, we acknowledge that it may not fully capture the complexity of personality traits. Future studies should employ validated instruments such as the Eysenck Personality Questionnaire or the Big Five Inventory to address this limitation.

Fifth, recruitment through an online questionnaire platform may have introduced selection bias, as participation could differ by education, socioeconomic status, internet access, and health awareness. Although the two-stage quota sampling strategy was designed to approximate the national distribution of the population by region and sex, the resulting sample was predominantly urban and relatively highly educated, which may limit the generalizability of our findings to the broader Chinese male population. In addition, our cohort primarily consisted of individuals of Han Chinese ethnicity, which may further restrict generalizability to other demographic groups.

## 5 Conclusion

Our findings suggest that exposure to extremely high temperatures and higher DTR during adolescence may be associated with higher PEDT scores in adulthood, whereas no association was observed with IIEF-5 scores. Given the cross-sectional design and the retrospective reconstruction of adolescent exposures, these findings should be regarded as preliminary and require confirmation in prospective longitudinal studies.

## Data Availability

The raw data supporting the conclusions of this article will be made available by the authors, without undue reservation.
