# Supplementary material for: Association of ambient temperature and diurnal temperature range exposure during adolescence with adult IIEF-5 and PEDT scores: a cross-sectional study in China
Source: Front Reprod Health. 2026 Oct 16;8:1955542. doi: 10.3389/frph.2026.1955542 (PMC13719567; doi:10.3389/frph.2026.1955542)
Supplement: Supplementary file 2 [file Table1.docx]

Contents

Supplementary Figure 1: Directed Acyclic Graph of this study.

Supplementary Figure 2: Box plot representation of the average levels for temperature and DTR measured during all, early, and late adolescence.

Supplementary Figure 3: Curves of the associations between IIEF-5 scores and average temperature and DTR exposure.

Table S1. Percentile-based cut-off values for extreme temperature exposure by region and exposure period.

Table S2. Tests for departure from linearity in the exposure-response relationships between adolescent temperature/DTR exposure and IIEF-5 and PEDT scores.

Table S3. Associations between extreme temperature and DTR exposure and IIEF-5 and PEDT scores.

Table S4. Sensitivity analyses of the associations between extreme temperature and DTR exposure during adolescence and PEDT and IIEF-5 scores, excluding adult post-exposure covariates.

Table S5. Sensitivity analyses of the associations between extreme temperature and DTR exposure during adolescence and PEDT and IIEF-5 scores, additionally adjusting for adolescent NDVI and air pollutants.

Supplementary Figure 1: Directed Acyclic Graph of this study. This graph represents the hypothesized relationships among various factors related to male sexual function. Each node in the graph represents a specific variable: “Demographics (Age, Population, Residence, Region, and Income)”, “Lifestyle (BMI, smoking status, and drinking status)”, “Personal Characteristics (Personality)”, “Health Status (Constitution and underlying disease)”, “Psychological Status (Depression score)”, “Temperature”, “DTR”, and “Male Sexual Function”.

Supplementary Figure 2: Box plot representation of the average levels for temperature and DTR measured during all, early, and late adolescence.

Supplementary Figure 3: Curves of the associations between IIEF-5 scores and average temperature and DTR exposure. The potential nonlinear effects of mean temperature (A, B, and C), and DTR (D, E, and F) during adolescence on IIEF-5 scores are quantified using multi-variable GAM. The models were adjusted for age, population, residence, region, income, BMI, smoking status, alcohol consumption status, personality, constitution, underlying disease, depression score, temperature, NDVI and air pollutants (PM_2.5_, PM_10_, SO_2_, NO_2_, O_3_, and CO) during the 1 month before the survey, as well as temperature during the corresponding adolescence exposure period (only in DTR models). The vertical axis indicates partial effects on IIEF-5 scores. Solid black lines correspond to points estimate of β of IIEF-5; Shaded areas correspond to 95% confidence intervals.

Table S1. Percentile-based cut-off values for extreme temperature exposure by region and exposure period.

| Region | Exposure period | 10th percentile (°C) | 90th percentile (°C) |
| --- | --- | --- | --- |
| National | All adolescence | 10.78 | 22.61 |
|  | Early adolescence | 10.71 | 22.54 |
|  | Late adolescence | 10.73 | 22.65 |
| North | All adolescence | 6.51 | 15.07 |
|  | Early adolescence | 6.53 | 15.03 |
|  | Late adolescence | 6.49 | 15.19 |
| South | All adolescence | 16.02 | 22.95 |
|  | Early adolescence | 15.85 | 22.92 |
|  | Late adolescence | 16.09 | 22.99 |

Note: National thresholds were used in the main analysis, and region-specific thresholds were used in the north/south subgroup analyses. North: latitude ≥ 33°N; South: latitude < 33°N.

**Table S2. Tests for departure from linearity in the exposure-response relationships between adolescent temperature/DTR exposure and IIEF-5 and PEDT scores**

| Exposure |  | Exposure period | F | P for nonlinearity |
| --- | --- | --- | --- | --- |
| PEDT scores | Temperature | All adolescence | 5.72 | 0.02 |
|  |  | Early adolescence | 5.98 | 0.02 |
|  |  | Late adolescence | 5.10 | 0.03 |
|  | DTR | All adolescence | 4.47 | 0.04 |
|  |  | Early adolescence | 3.51 | 0.06 |
|  |  | Late adolescence | 5.23 | 0.02 |
| IIEF-5 scores | DTR | All adolescence | 1.78 | 0.18 |
|  |  | Early adolescence | 1.89 | 0.17 |
|  |  | Late adolescence | 1.78 | 0.18 |

Note: Departure from linearity was tested by comparing a linear model to a model with a penalized smooth term (s(x, k=3)) using an F-test. All models were adjusted for the same covariates as in the main analyses. For DTR models, temperature during the corresponding exposure period was included as a covariate. P < 0.05 indicates statistically significant departure from linearity. Non-linearity tests for IIEF-5 and temperature were not performed because no association was observed in the main analyses.

Table S3. Associations between extreme temperature and DTR exposure and IIEF-5 and PEDT scores.

|  | Adolescence periods | Temperature exposure | | | Quintile of DTR exposure | | | | |
| --- | --- | --- | --- | --- | --- | --- | --- | --- | --- |
|  |  | Extremely cold | Moderate | Extremely hot | Q1 | Q2 | Q3 | Q4 | Q5 |
| PEDT scores | All adolescence | 0.02(-0.48,0.53) | 0(ref) | 0.30(-0.13,0.73) | 0(ref) | 0.08(-0.24,0.40) | 0.26(-0.07,0.59) | 0.03(-0.36,0.42) | 0.75(0.29,1.22) * |
|  | Early adolescence | 0.00(-0.51,0.50) | 0(ref) | 0.67(0.24,1.10) * | 0(ref) | 0.17(-0.15,0.49) | 0.23(-0.10,0.56) | 0.18(-0.21,0.57) | 0.75(0.28,1.21) * |
|  | Late adolescence | 0.12(-0.38,0.63) | 0(ref) | 0.26(-0.17,0.69) | 0(ref) | 0.08(-0.24,0.40) | 0.14(-0.18,0.47) | -0.03(-0.41,0.35) | 0.48(0.00,0.96) * |
| IIEF-5 scores | All adolescence | -0.02(-0.53,0.49) | 0(ref) | 0.07(-0.36,0.50) | 0(ref) | 0.07(-0.25,0.39) | -0.03(-0.36,0.30) | 0.04(-0.35,0.43) | -0.12(-0.59,0.35) |
|  | Early adolescence | 0.14(-0.37,0.65) | 0(ref) | -0.24(-0.66,0.19) | 0(ref) | 0.07(-0.25,0.39) | -0.06(-0.39,0.27) | 0.05(-0.34,0.44) | -0.02(-0.48,0.45) |
|  | Late adolescence | 0.06(-0.45,0.56) | 0(ref) | 0.08(-0.35,0.51) | 0(ref) | -0.13(-0.45,0.19) | -0.05(-0.37,0.28) | -0.07(-0.46,0.31) | -0.18(-0.65,0.29) |

Note: The reference temperature was moderate temperature (10th-90th percentile), and the reference DTR was Q1 (< 20th percentile). The 10th and 90th percentile cut-offs were 10.78℃ and 22.61℃ for all adolescence, 10.71℃ and 22.54℃ for early adolescence, and 10.73℃ and 22.65℃ for ate adolescence. Model adjusted for age, population, residence, region, income, BMI, smoking status, alcohol consumption status, personality, constitution, underlying disease, depression score, temperatures, NDVI and air pollutants (PM_2.5_, PM_10_, SO_2_, NO_2_, O_3_, and CO) during the 1 month before the survey, as well as temperature during the corresponding adolescence exposure period (only in DTR models). Black stars denote P < 0.05. Q1-Q5: quintiles.

Table S4. Sensitivity analyses of the associations between extreme temperature and DTR exposure during adolescence and PEDT and IIEF-5 scores, excluding adult post-exposure covariates

|  | Adolescence periods | Temperature exposure | | | Quintile of DTR exposure | | | | |
| --- | --- | --- | --- | --- | --- | --- | --- | --- | --- |
|  |  | Extremely cold | Moderate | Extremely hot | Q1 | Q2 | Q3 | Q4 | Q5 |
| PEDT scores | All adolescence | -0.05 (-0.59, 0.49) | 0(ref) | 0.50 (0.05, 0.96)* | 0(ref) | 0.05 (-0.29, 0.39) | 0.21 (-0.13, 0.55) | 0.05 (-0.36, 0.46) | 0.83 (0.34, 1.33)* |
|  | Early adolescence | -0.04 (-0.58, 0.50) | 0(ref) | 0.79 (0.33, 1.24)* | 0(ref) | 0.05 (-0.29, 0.39) | 0.14 (-0.20, 0.49) | 0.16 (-0.25, 0.57) | 0.76 (0.26, 1.25)* |
|  | Late adolescence | 0.08 (-0.46, 0.61) | 0(ref) | 0.43 (-0.03, 0.88) | 0(ref) | 0.01 (-0.33, 0.34) | 0.08 (-0.26, 0.42) | 0.01 (-0.39, 0.41) | 0.79 (0.30, 1.28)* |
| IIEF-5 scores | All adolescence | 0.00 (-0.53, 0.53) | 0(ref) | -0.12 (-0.57, 0.33) | 0(ref) | 0.08 (-0.25, 0.42) | 0.01 (-0.33, 0.35) | 0.03 (-0.37, 0.44) | -0.20 (-0.69, 0.29) |
|  | Early adolescence | 0.14 (-0.40, 0.67) | 0(ref) | -0.36 (-0.80, 0.09) | 0(ref) | 0.15 (-0.18, 0.48) | 0.01 (-0.33, 0.35) | 0.08 (-0.33, 0.48) | -0.03 (-0.52, 0.46) |
|  | Late adolescence | 0.05 (-0.48, 0.58) | 0(ref) | -0.08 (-0.53, 0.38) | 0(ref) | -0.09 (-0.42, 0.24) | 0.02 (-0.31, 0.36) | -0.06 (-0.46, 0.34) | -0.28 (-0.77, 0.20) |

Note: The reference temperature was moderate temperature (10th-90th percentile), and the reference DTR was Q1 (< 20th percentile). The 10th and 90th percentile cut-offs were 10.78℃ and 22.61℃ for all adolescence, 10.71℃ and 22.54℃ for early adolescence, and 10.73℃ and 22.65℃ for late adolescence. Models were adjusted for age, population, residence, region, income, personality, constitution, temperatures, NDVI and air pollutants (PM_2.5_, PM_10_, SO_2_, NO_2_, O_3_, and CO) during the 1 month before the survey, as well as temperature during the corresponding adolescence exposure period (only in DTR models). Sensitivity analyses excluded adult post-exposure covariates (BMI, depression, underlying disease, smoking status, and alcohol consumption status). Black stars denote P < 0.05. Q1-Q5: quintiles.

Table S5. Sensitivity analyses of the associations between extreme temperature and DTR exposure during adolescence and PEDT and IIEF-5 scores, additionally adjusting for adolescent NDVI and air pollutants.

|  | Adolescence periods | Temperature exposure | | | Quintile of DTR exposure | | | | |
| --- | --- | --- | --- | --- | --- | --- | --- | --- | --- |
|  |  | Extremely cold | Moderate | Extremely hot | Q1 | Q2 | Q3 | Q4 | Q5 |
| PEDT scores | All adolescence | -0.05(-0.70,0.60) | 0(ref) | 0.22(-0.28,0.73) | 0(ref) | 0.17(-0.21,0.54) | 0.29(-0.09,0.66) | 0.16(-0.30,0.63) | 0.67(0.10,1.23) * |
|  | Early adolescence | -0.14(-0.79,0.52) | 0(ref) | 0.63(0.13,1.13) * | 0(ref) | 0.14(-0.23,0.52) | 0.32(-0.05,0.70) | 0.46(-0.01,0.93) | 0.87(0.32,1.42) * |
|  | Late adolescence | 0.09(-0.55,0.73) | 0(ref) | 0.31(-0.19,0.82) | 0(ref) | 0.19(-0.18,0.55) | 0.20(-0.17,0.58) | 0.17(-0.29,0.62) | 0.81(0.27,1.35) * |
| IIEF-5 scores | All adolescence | 0.23(-0.44,0.91) | 0(ref) | 0.10(-0.43,0.62) | 0(ref) | 0.11(-0.28,0.49) | -0.09(-0.48,0.30) | 0.00(-0.48,0.48) | -0.18(-0.75,0.39) |
|  | Early adolescence | 0.44(-0.23,1.12) | 0(ref) | -0.32(-0.84,0.20) | 0(ref) | 0.10(-0.29,0.49) | -0.07(-0.47,0.32) | 0.00(-0.49,0.49) | -0.04(-0.62,0.53) |
|  | Late adolescence | 0.25(-0.41,0.91) | 0(ref) | 0.11(-0.42,0.63) | 0(ref) | -0.11(-0.49,0.27) | -0.06(-0.45,0.32) | -0.18(-0.65,0.30) | -0.22(-0.78,0.34) |

Note: The reference temperature was moderate temperature (10th-90th percentile), and the reference DTR was Q1 (< 20th percentile).

Models were adjusted for age, population, residence, region, income, BMI, smoking status, alcohol consumption status, personality, constitution, underlying disease, depression score, temperatures, NDVI and air pollutants (PM_2.5_, PM_10_, SO_2_, NO_2_, O_3_, and CO) during the 1 month before the survey, as well as temperature during the corresponding adolescence exposure period (only in DTR models). This sensitivity analysis additionally adjusted for NDVI and air pollutants during each adolescence exposure period. Black stars denote *p* < 0.05. Q1-Q5: quintiles.
